# Supplementary material for: Are carbon emissions trading and green financial instruments synergistic? -Comprehensive quantitative research based on content analysis
Source: PLoS One. 2024 Mar 7;19(3):e0298601. doi: 10.1371/journal.pone.0298601 (PMC10919601; doi:10.1371/journal.pone.0298601)
Supplement: S1 Data — (ZIP) [file pone.0298601.s001.zip › Data/地级市Z_F工作报告.zip!/░▓╗╒/╕╖╤⌠╩╨/└┤╫╘╣1⁄2╓┌║┼í╛┬φ┐╦╩2╛▌═°í┐_2010.docx]

各位代表：

现在，我代表市人民政府，向大会作政府工作报告，请予审议，并请市政协委员和其他列席人员提出意见。

一、2009年工作回顾

2009年，面对国际金融危机的严峻挑战，全市人民在中共阜阳市委的正确领导下，深入学习实践科学发展观，同心同德，克难奋进，扎实做好保增长、保民生、保稳定各项工作，较好地完成了市三届人大四次会议确定的主要目标任务。初步核算，全市生产总值突破600亿元，达到607.8亿元，比上年增长12.8%；实现财政收入64.3亿元，增长26.7%，增幅居全省第四位；城镇居民人均可支配收入12693元，农民人均纯收入3519元，分别增长8.2%和10.4%。

一年来，主要做了以下六个方面的工作：

（一）突出第一要务，千方百计保增长。坚持把保增长作为经济工作的首要任务，认真贯彻落实中央和省应对金融危机的各项政策，出台了保增长的45条具体措施，国民经济连续6年保持两位数增长，人均生产总值迈上1000美元台阶。坚持把保工业、保企业作为保增长的重中之重，大力实施工业突破四年行动计划，先后召开了3次工业园区发展现场会和6次银企对接会，认真落实“五缓四降三补贴”等帮扶企业政策措施，工业经济速度效益同步提高，园区建设呈现加速发展态势。全市规模以上工业实现增加值155.1亿元，增长26.7%；实现利税83亿元，其中利润45.7亿元，分别增长38.6%和37.4%。工业用电量增长24.1%，居全省第一位。新增规模工业企业170家、亿元企业12家，产值超过10亿元的企业达到16家。工业化率达到30.2%，三次产业结构调整为28.8:35.9:35.3，二产上升为第一大产业。全民创业行动扎实开展，全年新增个体工商户3.07万户。坚持把强农业作为保增长的重要基础，深入推进加快现代农业四年提升行动，战胜了50年一遇的特大旱灾，粮食生产连续六年获得丰收，总产再创历史新高，达到103.1亿斤，“江淮粮仓”地位进一步巩固。养殖业健康发展，万头养猪场、千头养牛场分别达到32个和21个，规模养殖比重达到60%，比上年提高5个百分点。新增市级以上龙头企业58家、农业标准化基地13个、农民专业合作组织334个，国家级大豆、甘薯、芝麻技术体系综合试验站在我市挂牌。集体林权制度主体改革全面完成，农业机械化水平进一步提高。坚持把促消费作为保增长的重要支撑，大力实施现代商贸物流四年提升行动，扎实推进“万村千乡”市场工程和“新网工程”，认真开展家电和汽车摩托车下乡活动。开工建设了阜阳临沂商城、汽贸物流园和香港财富广场。城乡市场繁荣活跃，实现社会消费品零售总额282.2亿元，增长18.7%。全市外贸进出口总额突破2亿美元，达到2.1亿美元，增长13.6%，增幅居全省第四位。金融保险业稳健运行，年末全市金融机构存款余额860.5亿元，贷款余额373亿元，分别增长18.4%和20.6%。

（二）突出扩大投资，多措并举上项目。坚持把扩大投资作为保增长的主要抓手，抢抓中央扩内需政策机遇，扎实做好项目谋划争取工作，深入实施“861”行动计划和“6611”工程，全市项目储备规模达3000亿元，累计争取中央新增投资项目718个，资金8.5亿元；完成固定资产投资250.5亿元，增长32.6%。其中城镇50万元以上项目投资181.5亿元，增长44%；全年新开工项目1150个，增长50%。重点项目建设取得积极进展，京九铁路电气化改造、阜周高速、沙颍河近期治理工程和濛洼中岗大桥建设顺利完成，口孜东煤矿、漯阜铁路复线电气化和阜阳机场提升通航保障能力改造工程建设步伐加快，阜六铁路、阜新高速、阜阳船闸和金种子集团优质白酒扩容改造、昊源集团和临化公司新增140万吨尿素、阜淮铁路电气化改造项目开工建设，开乐公司新增万辆罐式专用车、界首至临泉铁路等项目前期工作进展顺利。与央企对接合作工作取得明显成效，共签约项目27个，总投资额达532.9亿元，居全省第三位。成功举办了第三届皖西北投资贸易洽谈会、第二届华药会和首届中国专用车产业发展高层论坛，组织参加了中博会、徽商大会、深圳高交会等重大经贸活动，与临沂市结为友好城市，与合肥市签订了结对合作框架协议。全年引进市外项目1008个，实际到位资金153.9亿元，增长36.9%。实际利用外资7006万美元。

（三）突出城乡建设，增加投入夯基础。坚持把加快城镇化进程作为保增长的重要举措，紧紧围绕建设皖西北中心城市和滨水园林城市，认真组织实施阜城总体规划，努力加大基础设施建设力度，累计完成投资13.08亿元。高标准改造了阜蚌路、阜涡路等5条出口路，北三环路建设、向阳北路北延、西城墙路改造工程竣工，颍河大桥主体工程基本完工，南京路、颍州路和阜王路南延工程加快建设，三清路网、七渔河路网和柳林路网工程前期工作进展顺利。沟河塘治理和美化、亮化工程扎实推进，三角洲公园和泉河景观带一期工程顺利完成，东城河、二里井河清淤疏浚工程完工，一道河治理工程加快实施，河滨路、西二环路及北京东路等道路绿化、亮化工程相继完工。实施了城市供水管网改造，中水利用工程和颍东污水处理厂加快建设，阜阳城市生活垃圾卫生填埋场建成使用，小街巷、城市公厕改造步伐加快，天然气管网和加气站建设稳步推进。扎实开展“五城同创”和省级文明城市创建活动，取缔了阜城非法客运电动三轮车，交通秩序明显改善。城乡爱国卫生运动扎实开展，全省爱国卫生运动现场会在我市召开。县城和小城镇建设同步发展，扩权强镇试点工作顺利开展，新增省级新农村建设示范村80个。

（四）突出以人为本，尽心尽力惠民生。坚持把保民生作为保增长的根本目的，大力实施28项民生工程，累计投入资金28.4亿元，其中市、县投入4.51亿元，惠及800多万城乡居民，荣获2009年全省民生工程组织实施工作杰出奖。进一步完善社会保障体系，加强就业再就业工作，认真解决高校毕业生、农民工和城镇困难群体就业问题，组织开展返乡农民工招聘活动，稳步推进失业和工伤保险市级统筹，妥善解决了困难企业职工养老和医疗保险问题，基本养老、基本医疗、失业、工伤、生育保险参保人数均超额完成省下达的目标任务。被征地农民社会保障制度全面实施，农村新型养老保险试点工作扎实开展。开工建设了廉租住房，发放了廉租住房补贴。深入推进科教兴阜战略，组织实施省级以上科技计划57项，全年专利申请量达到325件。义务教育阶段学校办学标准化、农村学区和职教园区建设得到加强，阜城中小学布点规划加快实施。基层医药卫生体制综合改革试点工作顺利启动，城乡卫生服务体系加快建设，建成乡镇卫生院19所、村卫生室407个，荣获全国无偿献血先进城市称号。全民健身运动广泛开展，市体育中心运动场建成使用。成功举办了市第11届运动会，阜阳籍运动员邓琳琳获得第41届世界体操锦标赛和第11届全运会体操金牌。低生育水平整体保持稳定，人口出生率控制在13.06‰，低于省下达指标。成品油价格和税费改革顺利完成，政府还贷二级公路收费全部取消，稳妥解决了工商系统市场办管脱钩问题。文化事业加快发展，广播电视“村村通”工程基本完成。城市社区换届工作顺利开展。第二次全国经济普查工作顺利完成，第二次全国土地调查和第三次全国文物普查工作扎实推进。大力弘扬“王家坝精神”、“农民工精神”，精神文明创建活动深入开展。老龄和老干部工作得到进一步加强。妇女、儿童及残疾人权益得到保障。新闻出版、海关、物价、外事、侨务、审计、统计、文物保护、档案、地方志、地名等工作也都取得了新的成绩。

（五）突出和谐发展，全力以赴保稳定。坚持把维护社会稳定作为第一责任，深入推进平安阜阳创建活动，组织开展扫黑除恶等专项斗争，严厉打击各种违法犯罪活动，圆满完成国庆60周年等重大活动安保任务，维护了社会治安大局稳定。坚持领导开门接访、带案下访和领导包案制度，扎实开展信访积案化解年活动，认真做好矛盾纠纷排查调处工作，信访秩序继续好转。建立健全应急机制，完善应急预案体系，严格落实安全生产责任制，扎实推进产品质量和食品药品安全监管，切实做好甲型H1N1流感、手足口病等传染病防治工作。生态阜阳建设稳步推进，节能减排年度工作任务顺利完成，颍州西湖、太和沙颍河列入国家湿地公园。扎实推进违法用地清理整治，土地管理工作进一步规范。全民国防教育和双拥共建活动广泛开展，荣获省级双拥模范城称号。武警、消防、人防和民兵预备役工作进一步加强。民族、宗教、对台、地震、气象、出入境检验检疫等工作也都取得了新进展。

（六）突出自身建设，创新管理提效能。坚持把强化自身建设作为保增长的重要保障，大力加强服务型政府建设，深入推进依法行政，实施了市、县政府机构改革。认真开展深入学习实践科学发展观活动，扎实推进作风建设年活动，组织开展了民主考评市直单位科长活动，着力提升工作效能。市、县行政服务中心建设进一步加强，实现了县、乡、村为民服务全程代理全覆盖。深入实施《政府信息公开条例》，切实加快信息化步伐，全面启动电子政务二期工程，农村综合信息服务试点工作通过国家验收。认真贯彻落实中央关于党政机关厉行节约的八项要求，加强中央扩内需保增长政策落实情况的监督检查，开展了清理“小金库”和工程建设领域突出问题专项治理工作。坚持从严治政，强化行政监察和审计监督，扎实推进政风和行风建设，惩治和预防腐败体系不断完善。各级政府自觉接受人大法律监督和政协民主监督，认真办理人大代表议案、建议和政协委员提案，广泛听取各民主党派、工商联、无党派人士及各人民团体的建议和意见，鼓励支持人民群众、新闻媒体对政府工作进行监督，政府工作透明度和社会公众参与度进一步提高。

在肯定成绩的同时，我们也清醒地认识到，我市经济社会发展中还存在一些不容忽视的困难和问题。一是从经济发展看，尽管全市生产总值连续6年保持两位数增长，但经济总量依然偏小，人均水平与全省相比仍有较大差距，实现“十一五”规划“两个确保”目标任务依然艰巨。二是从产业结构看，尽管我市经济结构逐步得到改善，但工业化、城镇化水平依然偏低，现代服务业发展滞后，县域经济总体实力较弱，园区载体作用有待进一步增强。三是从投资需求看，尽管固定资产投资增速加快，但投资总量依然偏小，招商引资工作仍处于低水平状态，大项目好项目还比较少。四是从制约要素看，尽管在破解要素制约方面采取了一些措施，但资金制约还没有得到有效缓解，存贷比依然偏低，用地不足的矛盾仍然突出，实现“十一五”节能减排目标还需付出艰辛努力。五是从和谐发展看，尽管民计民生有了改善，但与人民群众的期望还有不少差距，重点群体就业压力较大，社会保障体系不够健全，城乡居民收入偏低，社会建设仍很薄弱，社会热点难点问题和不稳定因素依然较多。六是从政府工作看，尽管机关效能得到进一步提高，但政府职能转变与科学发展的要求还不完全适应，工作作风不实、执行落实不力、办事效率不高以及不作为、乱作为、慢作为的问题仍很突出，对群众反映的热点、难点问题解决还不够及时，形式主义、官僚主义、铺张浪费甚至贪污受贿等腐败现象还不同程度地存在。对此，我们一定高度重视，采取切实有效措施，认真加以解决。

各位代表，过去的一年，在面对诸多不利因素的情况下，我们能够取得经济社会发展的重大成就，确实来之不易。这是省委、省政府和市委正确领导的结果，是市几套班子大力支持的结果，是全市广大干部群众齐心协力、共克时艰的结果。在此，我代表市人民政府，向辛勤工作在全市各条战线上的广大干部群众，向给予政府工作大力支持与监督的人大代表、政协委员、民主党派、工商联、无党派人士、各人民团体、新闻媒体和其他各界人士，向关心支持阜阳建设的离退休老干部、老职工，向为阜阳改革发展稳定作出贡献的解放军指战员、武警消防官兵、预备役部队、政法干警，向中央和省驻阜单位及所有关心支持阜阳建设与发展的同志们、朋友们，向在我市创业的境内外投资者、建设者，致以崇高的敬意和衷心的感谢！

二、2010年工作安排

2010年是实施“十一五”规划的最后一年，也是巩固经济回升基础、谋划“十二五”发展的关键一年。我们既面临着中部崛起战略深入实施、东部沿海地区产业加速转移和省委、省政府支持皖北发展等众多机遇，也面临着经济发展滞后、结构矛盾突出、区域竞争激烈等诸多困难和挑战。但总体上机遇大于挑战，我们已经站在了一个新的历史起点上，到了应该加速发展也能够加速发展的关键时期。只要全市上下振奋精神、坚定信心，抢抓机遇、迎难而上，开拓进取、扎实工作，就一定能够夺取科学发展、加速崛起的新胜利，开创阜阳经济社会发展的新局面。

今年政府工作的指导思想是：以邓小平理论和“三个代表”重要思想为指导，深入贯彻落实科学发展观，着力促进发展方式转变和经济结构调整，切实加快工业化和城镇化进程，大力发展现代农业和商贸物流业，强力推进项目建设和招商引资，继续加强资源节约和环境保护，更加重视改善民生和促进社会和谐，全面提升经济发展规模和水平，全力推动经济社会又好又快发展。

综合各种因素考虑，今年经济社会发展的主要目标是：全市生产总值增长12%以上，财政收入增长20％以上，固定资产投资增长30％以上，招商引资增长30％以上，社会消费品零售总额增长15％以上，进出口总额增长15％以上，城镇居民人均可支配收入增长8％，农民人均纯收入增长8％，人口自然增长率控制在8.5‰以内，城镇登记失业率控制在4％以内，全面完成节能减排“十一五”目标任务。

围绕上述目标，重点抓好以下八个方面的工作：

（一）围绕做大做强，进一步加大主攻工业力度。深入实施工业突破四年行动计划，全面加快工业化进程，确保全年工业投资增长45%以上、规模以上工业增加值增长25%以上。一是着力壮大支柱产业。加大重点产业和骨干企业的扶持力度，突出抓好煤电、化工、食品、有色金属、机械制造、林产品、生物医药和纺织服装八大支柱产业，力争全年工业产值突破700亿元。围绕打造煤电煤化工基地，加快推进煤电一体化进程，重点支持刘庄煤矿和谢桥煤矿扩大生产，确保口孜东煤矿及早建成投产，力争罗园煤矿和口孜西煤矿开工建设、阜阳华润电厂二期项目获得国家批准。大力推进煤电联产和煤炭精深加工，继续做好刘庄坑口电厂、矸石电厂和颍东口孜电厂项目前期工作，力争煤电工业产值突破150亿元。支持昊源集团和临化公司扩大生产能力，加快推进颍上鑫泰和阜南新天化工公司建设，力争化学工业产值突破100亿元。围绕打造农副产品加工基地，加快金种子集团优质白酒扩容改造和雪花啤酒年产35万吨扩建项目建设，力争食品工业产值突破150亿元。以沪千集团、大亚集团、东盾木业、鹏程文教等企业为龙头，延伸林产品加工产业链条，提高产品附加值，做大林产品加工业。以界首田营、太和肖口等有色金属产业园为依托，大力推广铅、铝冶炼环保节能新技术，加快电解铝、蓄电池和铅化工项目建设，不断延伸产业链条，力争有色金属工业产值突破150亿元。围绕打造专用车产业基地，积极整合产业资源，加快开乐公司新增万辆罐式专用车项目建设，大力发展汽车、农机、通用机械等装备制造业。依托阜阳和太和医药配送中心，以市场带企业，重点扶持锦辉、华信、仁济、贝克药业等龙头企业，发展壮大生物医药产业。组织实施华源纺织退城进园，培育壮大京九丝绸、皓辉纺织、青岛即发、三宝线业等龙头企业，做大纺织服装业。二是切实加强改革创新。进一步加大技术改造力度，重点抓好174个投资1000万元以上的技改项目，力争昊源集团和临化公司新增140万吨尿素、悦康药业年产2000吨头孢类原料、京九丝绸年产800万米新型高档真丝面料等重点项目建成投产。大力扶持具备条件的企业上市融资，认真做好上市企业后备资源培育工作，努力扩大直接融资规模。加快培育生物制药、电子信息、节能环保等新兴产业和高新技术产业，推进产业优化升级。进一步深化企业改革，扎实推进阜阳轴承、青峰机械等企业资产重组工作，积极引进战略投资者，多渠道筹集改革资金，加快推进企业改制进程。切实加强企业家队伍建设，着力营造有利于企业家干事创业的良好氛围。三是加快园区建设步伐。认真落实加快工业园区发展的政策措施，加大资金投入，加强协调调度，促进工业园区快速发展。大力支持阜阳经济技术开发区加快扩区步伐，发展优势产业和高新技术产业，力争完成基础设施投入4亿元以上、建成区面积扩大4平方公里以上。大力推进县域工业园区和乡镇工业聚集区建设，培育壮大一批专精特新中小企业，着力构筑特色经济和产业集群。大力发展非公有制经济，放宽市场准入，降低创业门槛，激活民间投资，努力形成全民创业的局面。四是突出抓好节能降耗工作。严格落实节能降耗目标责任制，加强年耗能3000吨标煤以上重点用能单位的监管，引导企业加大节能降耗投资，实施节能十项攻坚。大力发展循环经济和低碳经济，推进高碳产业低碳化改造和低碳技术应用，努力实现可持续发展。

（二）围绕扩容提质，进一步加快城镇建设步伐。以建设皖西北中心城市和滨水园林城市为目标，全面加快城镇化进程，强力推进阜城大建设大发展，力争全年基础设施投入达到40亿元以上。一是拓展城市发展空间。以路网建设为支撑，进一步扩大阜城规模，重点实施三清路网、七渔河路网和柳林路网建设工程，完成阜临路七渔河至颍州西湖段道路改造，加快城南新区和泉北新区、颍东新区建设，推进南京路、北京路、河滨路、莲花路等道路延伸工程，完成东三环路建设的前期工作。实施阜城6条出口道路两侧和城区道路综合改造工程，改造清河路、河滨南路、阜王路、北京中路，打通双清路东段、东城墙路北段和临泉路西段。加快颍上路泉河大桥、颍河船闸桥和北三环路下穿铁路立交工程建设进度，建设颍州路与文峰路交叉口人行天桥。二是完善城市整体功能。大力实施城市道路和园林景观绿化、亮化、美化工程，加强颍河、泉河城区段环境综合治理，高标准建设泉河北岸风景带，实施三角洲公园二期工程。加快西城河风景带和环城河景观带建设，开展七渔河、一道河、七里长沟等沟河塘环境治理。加快小街巷改造步伐，实施城区供水、污水管网改造和中水利用项目建设，建成颍东污水处理厂。加快水源地建设和泉北污水处理厂项目前期工作。大力改造棚户区和城中村，推进安置房、廉租房和经济适用房建设。进一步规范经营性土地出让和拆迁安置工作，促进房地产业快速健康发展。三是提升城市管理水平。完善阜阳城市总体规划，加快编制城市发展战略规划，做好淮河路城市设计。深入开展文明城市创建活动，加大“五城同创”力度，提升城市品位。创新城市管理理念，加大违法建设防控力度，切实解决脏、乱、差等突出问题。进一步规范城市交通秩序，巩固阜城非法客运电动三轮车整治成果，大力发展城市公共交通事业，适度增加出租车运力。四是加快城镇发展步伐。以县城为重点，加大投入力度，构筑县域经济发展的重要平台。继续实施“千村百镇”示范工程，扎实推进扩权强镇试点工作，完成市、县、乡土地利用总体规划修编工作，强力推进省级示范镇、中心镇和建制镇建设，增强城镇集聚力和辐射力。加强新农村建设规划引导，着力解决农民建房需求问题。深化户籍制度改革，认真落实中央放宽中小城市和小城镇落户条件的政策，不断扩大城镇人口规模。

（三）围绕增效增收，进一步夯实农业基础。坚持把解决好“三农”问题作为全部工作的重中之重，认真贯彻落实2010年中央1号文件精神，加大统筹城乡发展力度，深入实施加快现代农业四年提升行动，不断夯实农业农村发展基础。一是主攻粮食单产，努力增加总产。按照“稳定面积、主攻单产、提升品质、增加总产”的思路，深入推进粮食生产“三大行动”，扎实开展太和、界首小麦和临泉、颍州玉米单产千斤县创建活动，支持鼓励种粮大户开展规模经营，努力提高粮食单产和优质化率，增加粮食总产，巩固江淮粮仓地位。二是调整农业结构，提高经济效益。加快推进畜牧水产规模化、标准化健康养殖，实施水产跨越工程和水产良种工程，建设优质安全畜产品供给功能区和现代畜牧业科技示范区。大力发展高效经济作物，扩大蔬菜、中药材等种植面积，继续实施林业重点生态工程和万里绿色长廊工程，建设高标准农田林网示范区，抓好合淮阜和济广高速公路阜阳段绿化。扎实开展国家级、省级现代农业示范区创建活动，加快建设太和县东方现代农业等8个农业科技示范园。深入实施“安全优质农产品标准化示范工程”，实行农产品质量安全全程监控，推广农业标准化生产，加快建设国家粮油加工质检中心，争创国家级和省级标准化生产基地3个。三是培育龙头企业，提升产业化水平。进一步推进农业产业化提升行动，加强资金、技术和人才支持，大力扶持农业产业化龙头企业，积极引进优势企业，壮大龙头企业实力。强化市场和利益调节，创新经营体制，鼓励发展农民专业合作组织，引导农民开展资金互助和土地股份合作。支持阜南黄岗、界首陶楼等25个省级特色示范村镇加快发展，完成186个市级以上特色专业村产业发展规划。四是壮大劳务经济，促进农民增收。进一步加强农民工培训工作，扎实开展职业技能培训和创业培训。积极搭建劳务对接平台，拓展海外劳务输出业务，提高组织化输出水平。引导农民向二、三产业和城镇转移，增加工资性收入。深入实施“凤还巢”工程，加快农民工创业园区建设，扶持返乡农民工入园创办或领办企业。五是强化基础设施，提高综合生产能力。加强中低产田改造，建设一批高标准农田和基本农田保护示范区。抓住国家开展小型农田水利重点县建设的机遇，加强农田水利建设，实施中小河流综合治理项目。完善现代农业产业技术体系和基层农技推广体系，建设13个国家和省级现代农业综合实验站。扎实推进农业机械化，提高农机装备水平。深入推进农村饮水安全、“村村通”延伸工程和电力基础设施建设。加快推广沼气、秸秆综合利用等可再生能源技术。六是深化农村改革，增强农村发展活力。巩固农村综合改革成果，稳定农村基本经营制度。在保持农村土地承包关系稳定的基础上，按照依法自愿有偿原则，鼓励农民以转包、转让、出租、互换、股份合作等多种形式流转农村土地承包经营权。完成农村集体土地所有权、宅基地及集体建设用地使用权等确权登记颁证工作，推进村级公益事业建设一事一议财政奖补试点。加强新农村综合示范区建设，总结推广农村土地整治整村推进试点经验，加快中心村建设和空心村改造步伐。

（四）围绕市场繁荣，进一步提升服务业水平。充分发挥综合区位交通优势，大力实施商贸流通百亿元工程和现代商贸物流四年提升行动，努力打造区域性商贸物流名城。一是推进核心商业区建设。大力培育国贸商城、百货大楼等商贸龙头企业，加快香港财富广场、商厦四期、和华千百意二期工程建设，提升人民路核心商业区档次。加快大润发、红星美凯龙等项目建设进度，构建西二环中心商业区。完善阜城商业网点，形成超市、名品店、大卖场综合体等高中低不同档次、不同业态并存的格局。二是增强市场辐射力。进一步做大做强阜阳和太和医药配送中心，加快阜阳临沂商城和阜阳汽贸物流园建设步伐，实施阜阳农产品中心批发市场迁建工程，改造提升皖西北商贸城，规划建设工程机械、农资农机、粮食食品等大型专业市场，着力培育一批辐射范围广、产业关联度高、带动能力强的专业市场，形成较大规模的现代市场集群。三是提升物流业发展档次。加强物流业基础设施建设，加快中储粮农产品物流园、阜阳粮食产业园、中石油油库等物流园建设步伐，完善提高华联、商厦、汽运、烟草等物流配送中心功能。积极发展服务外包、第三方物流等新型业态，努力构建层次分明、业态先进、功能完备的商贸物流体系。四是加快发展现代服务业。适应群众生活多样性、个性化需求，大力发展信息咨询、商务服务、保险、娱乐健身和社会化养老等现代新型服务业。深入实施“双百市场”、“农超对接”工程和“万村千乡”市场工程、“新网工程”，扎实推进家电下乡、汽车摩托车下乡及汽车、家电以旧换新工作，扩大政策覆盖面，服务农村消费，繁荣农村市场。进一步挖掘阜阳历史文化资源，加强旅游景区规划建设，着力提升颍州西湖、阜阳生态园、颍上八里河和迪沟风景区等旅游景点的档次。大力发展乡村旅游、生态旅游，增强阜阳旅游吸引力。五是努力扩大对外贸易。大力培育外向型企业，重点扶持人发、柳编、洁具、香料、脱水蔬菜、中药材、渔具等出口基地，加强海铁联运物流“无水港”建设，扩大出口规模。组织参加广交会、华交会等经贸活动，支持企业开拓国际市场，增加特色农产品和劳动密集型产品出口，提高经济外向度。

（五）围绕增强后劲，进一步扩大投资规模。初步安排，今年“6611”工程项目464个，年度计划投资194亿元，其中列入“861”行动计划项目190个，年度计划投资150亿元，力争全年固定资产投资350亿元以上。一是加大项目谋划争取力度。进一步完善项目谋划机制，瞄准国家投资导向和产业结构调整方向，紧紧围绕“三农”、科技创新、社会事业、民生工程和重大基础设施建设，不断扩大项目储备规模。加大项目经费投入，扎实做好前期工作，争取更多的项目落户阜阳。二是加大项目建设力度。继续实行市级领导联系重大项目制度，强化协调调度，及时解决项目建设中的矛盾和问题。加快阜新高速、阜六铁路建设和漯阜铁路复线、阜淮铁路电气化改造步伐，完成阜阳船闸主体工程，力争沿淮洼地治理、沙颍河航道整治、杨桥船闸等项目开工建设，界首至临泉铁路等项目得到核准。切实加强项目管理，强化资金监管，确保工程质量。大力开展土地复垦整理，扎实做好新增土地与建设用地置换挂钩工作，确保重点项目建设用地。加强建设用地监管，开展违法用地清理整治工作，提高土地使用效益。三是加大招商引资力度。抢抓沿海发达地区产业加速转移的机遇，完善以政府为导向、专业队伍为骨干、企业为主体的招商工作机制，广泛开展领导带头招商、以商招商、以企招商、产业招商，组织参加上海世博会和徽商大会等重大招商活动，办好第四届皖西北投资贸易洽谈会和具有地方特色的各类节会活动，吸引更多的客商来我市投资兴业。四是加大对接合作力度。扎实做好与央企对接合作成果的落实转化工作，大力推进已签约项目的跟踪落实，探索建立合作长效机制，不断拓展合作的深度和广度。进一步深化与合肥的结对合作，加大框架协议落实力度，细化工作措施，力求在产业升级、园区建设、企业引进、教育合作、人才培训等方面取得实质性进展。

（六）围绕保障有力，进一步做好财政金融工作。充分发挥财政金融杠杆作用，积极推动财政金融改革与创新，更好地支持和服务经济社会发展。一是强化财政保障能力。落实结构性减税政策，加大清费治乱力度，切实减轻企业负担。实施财源建设规划，发挥财政资金的引导和带动作用，培植壮大财源。依法加强税收征管和社保费征缴，规范税费收入管理。坚持集中财力办大事，调整优化财政支出结构，整合财政专项资金，发挥财政对转变发展方式、保障和改善民生的支持作用。深化部门预算、“收支两条线”管理、财政支出绩效评价改革，不断完善政府采购、国库集中支付制度，稳步推进全市财税库银横向联网工作，实现财政科学化精细化管理。加强各项财政性资金的审计监督，强化政府债务管理，规范财经秩序。二是着力提升信贷服务水平。认真落实与各金融机构签订的战略合作协议，完善政银企沟通合作机制。继续举办银企对接活动，做好企业债券发行和使用工作。大力推进金融产品和服务创新，开发适合中小企业特点和个体工商户、农户需求的信贷产品，增加信贷额度，提高存贷比，力争全年贷款余额突破500亿元。加强政府投融资平台建设，整合存量资产，规范资金运营管理，提高政府投融资平台的资本规模和信用等级，增强投融资能力。加大县域经济发展的支持力度，鼓励县域金融机构将新增贷款主要投在当地。三是加快地方金融体系建设。深化农村信用社改革，积极推动农信社组建为农村商业（合作）银行，大力发展村镇银行、小额贷款公司，促进证券、保险等行业健康发展，增强担保公司担保能力。积极引进股份制商业银行设立分支机构，实现金融服务乡镇全覆盖。打造诚信阜阳，创建信用村镇，营造良好金融生态。依法严厉打击非法集资、逃废金融债务等不法行为，着力规范金融市场秩序。

（七）围绕改善民生，进一步维护和谐稳定大局。坚持以人为本、民生为重，扎实做好就业和社会保障工作，切实维护社会稳定，建设和谐阜阳。一是拓展提升民生工程。进一步加大投入，将民生工程扩大为33项，新增中小学校舍安全工程、社会（儿童）福利中心、农村留守儿童之家、光荣院建设和农村清洁工程5项，提标扩面农村居民最低生活保障、城乡卫生服务体系建设、新型农村合作医疗等6项。完善民生工程实施和管理机制，加强协调调度，规范资金管理，切实提高工程质量和资金使用效益。二是加强就业再就业工作。加快健全覆盖城乡的公共就业服务体系，全面提高公共就业服务水平。继续执行“五缓四降三补贴”政策，多渠道增加就业岗位。加强就业和社会保障公共服务设施建设，加大技能培训、政策帮扶和就业服务援助力度，开发公益性岗位，重点解决高校毕业生、农民工和困难群体就业问题。鼓励支持劳动者就地就近就业，力争全年新增就业岗位5.4万个、下岗失业人员再就业1.4万人。加强劳动监察，做好劳动关系协调和争议调解仲裁工作，切实维护劳动者合法权益。三是加快完善社会保障体系。完善城镇职工养老、失业、医疗保险制度，全面实施被征地农民养老保险制度，积极稳妥推进新型农村社会养老保险试点。认真落实养老保险关系接续办法，实施城镇企业职工基本养老保险关系转移接续制度，保障流动就业人群权益。全面实施基本养老保险省级统筹，制定落实市级配套政策。实行工伤保险市级统筹，扩大医疗、失业保险市级统筹范围。完善社保救助体系，实施城乡低保对象、企业退休人员、农村五保户、部分优抚对象的扩面提标。四是全力抓好应急管理和安全生产。进一步完善应急管理体系，推进应急平台建设和力量整合，提高保障公共安全和处置突发事件的能力。严格落实安全生产责任制，大力开展交通、消防和建筑安全等专项整治，坚决遏制重特大事故的发生。切实做好工商行政管理、质量技术监督、出入境检验检疫和市场价格监管工作。建立健全食品药品安全监管长效机制，保障人民群众身体健康。五是深入推进平安阜阳建设。加强和改进信访工作，坚持领导干部开门接访、带案下访，规范信访秩序，解决人民群众反映的热点难点问题。健全社会舆情汇集和分析机制，完善利益表达、权益保障、矛盾调处机制，有效预防和妥善处置群体性事件。进一步落实维护社会稳定第一责任，深入推进扫黑除恶等各类专项斗争，加强社会治安综合治理，依法打击违法犯罪活动，增强人民群众的安全感。

（八）围绕协调发展，进一步促进人与社会全面进步。统筹经济社会协调发展，更加注重社会各项事业建设，着力提高社会管理和公共服务水平。一是深入实施创新推动战略。推进创新体系建设,加大科技投入，提升产学研合作水平。加强知识产权保护，促进高新技术成果产业化。实施人才强市战略，完善落实人才激励政策，大力引进人才智力，吸引更多的高层次、创新型人才来阜创业。二是优先发展教育事业。全面实施素质教育，健全义务教育经费保障机制，加快实施学校布局规划、标准化建设和校舍安全改造工程，促进义务教育均衡发展。加快普及高中阶段教育，重视学前教育，关心特殊教育，发展民办教育。进一步规范学校办学行为，加强教师队伍建设，提高教育教学质量。深入推进职教大市建设，整合职业教育资源，加快阜阳职教园区建设步伐。大力支持阜阳大学、阜阳技师学院、阜阳汽车技师学院和阜阳师范专科学校筹建工作。继续做好家庭经济困难学生资助工作，认真解决农村留守儿童就读和生活困难问题。三是加快发展公共卫生事业。进一步强化政府责任和财政投入，加强覆盖城乡居民的基本医疗卫生服务体系建设，筹建1所三甲标准的医院。完善农村三级卫生服务网络和城市社区卫生服务体系，增强基本公共卫生和基本医疗服务能力，切实缓解看病难、看病贵问题。加快实施国家基本药物制度，扎实推进基层医药卫生体制综合改革试点工作。探索公立医院改革，加快发展中医医疗服务体系。深入开展爱国卫生运动，继续做好艾滋病、甲型H1N1流感等重大疾病和传染病防治救助工作。完善新型农村合作医疗制度，提高筹资标准和补偿待遇，将参合农民的补助标准由年人均80元提高到120元。四是大力繁荣文化体育事业。加强社会主义核心价值体系建设，深入开展思想道德教育和群众性文明创建活动，大力弘扬“王家坝精神”、“农民工精神”，规划建设王家坝纪念馆、农民工纪念广场。全面推进文化体制改革，加强人才队伍建设，培育文化龙头企业，打造一批文艺精品。广泛开展群众性文化活动，完善公共文化服务体系，深入实施“文化惠民”工程。开工建设市体育中心综合馆，规划迁建市博物馆、图书馆、文化馆。加强阜阳特色文化资源开发利用工作，适时举办非物质文化遗产宣传推介活动，完成第三次全国文物普查和首次非物质文化遗产普查。深入开展“扫黄打非”，加强网络文化建设和管理，规范文化市场秩序。广泛开展全民健身运动，提高竞技体育水平。继续实施广播电视“村村通”、农家书屋和乡镇综合文化站建设，加快残疾人综合服务中心建设。五是加强人口和计划生育工作。认真落实计划生育责任制和责任追究制，突出抓好人口大县、后进乡村和流动人口的计划生育工作，扩大长效节育措施奖励扶持范围，综合治理出生人口性别比，提高计划生育服务质量，稳定低生育水平。六是加快环境友好型社会建设。坚持可持续发展战略，加快生态阜阳建设，推进污染减排工作，开展省级环保模范城区和环境优美乡镇、生态村、生态户创建活动，倡导节约型生产方式、消费模式和生活习惯，改善城乡人居环境。加强社区建设，着力构建和谐社区。强化统计基层基础工作，做好第六次全国人口普查、第一次全国水利普查和城镇住户大样本调查轮换工作。进一步加强法律援助和人民调解工作。巩固和发展平等团结互助和谐的民族关系，发挥宗教在促进社会和谐方面的积极作用。加强国家安全和保密工作。大力发展残疾人、红十字和福利慈善事业，切实保障妇女和未成年人权益。继续深化殡葬制度改革。支持驻阜部队建设，加强国防后备力量。深入推进“双拥”共建活动，巩固发展军政军民团结的良好局面。充分发挥老干部在构建和谐社会中的重要作用，支持关工委、老龄委和工会、共青团、妇联工作，继续加强新闻出版、海关、外事、对台、侨务、审计、档案、地名、地方志、地震、气象等各项工作。

各位代表，科学编制“十二五”规划，也是今年的一项重要工作。我们将以科学发展观为指导，立足阜阳实际，充分发扬民主，凝聚各方智慧，认真研究经济社会发展中的重大战略问题，切实增强规划的科学性、前瞻性和指导性，努力在更高水平上加速阜阳奋力崛起进程。

三、切实加强政府自身建设

扎实做好今年工作，完成“十一五”规划目标，对政府工作提出了新的更高的要求。我们必须进一步加快转变政府职能，切实提高行政效率和施政水平，更好地为改革发展大局服务，为广大人民群众服务。

（一）强化学习意识，增强跨越发展的坚定性。围绕建设学习型政府，各级政府及其工作人员要牢固树立重视学习、坚持学习、终身学习的理念，切实把学习作为提高素质、增强本领、做好工作的根本途径，努力使我们的思想适应形势发展、知识适应时代要求、能力适应工作需要。广泛学习履行职责必需的知识，更新完善知识结构，提高驾驭复杂局面、解决复杂问题的能力。深入开展向沈浩同志学习活动，大力弘扬牢记宗旨、爱岗敬业、淡泊名利、奋发进取的崇高精神，做人民满意的公务员。坚持理论联系实际的学风，自觉向群众学习、向基层学习、向实践学习，不断提高谋发展、破难题的能力。进一步解放思想、扩大开放，切实加快改革步伐，着力革除与时代要求不相适应的思维定势和工作方式，始终保持良好精神状态，做改革创新的促进派、科学发展的实践者，真正把解放思想的成效体现在攻坚克难上，把与时俱进的要求落实到具体工作中，努力推动阜阳经济跨越发展。

（二）强化法治意识，增强施政行为的规范性。围绕建设法治型政府，进一步规范执法行为，强化执法监督。继续推进行政审批制度改革，规划建设市招投标中心。坚持科学决策、民主决策、依法决策，完善政府重大事项决策公示制度、听证制度和专家咨询制度。进一步健全决策失误纠错改正机制和责任追究制度，开展政府工作绩效评估，做到有责必问、有错必纠。深入推进政务公开，完善新闻发布制度，加快电子政务建设，办好政府公报。切实加强政府法制工作，完善行政复议制度，全面完成“五五”普法任务。各级政府及其部门要自觉坚持党的领导，主动接受人大依法监督和政协民主监督，认真办理人大代表议案、建议和政协委员提案，虚心听取和采纳各民主党派、工商联、无党派人士及工会、共青团、妇联等社会团体的意见，主动接受社会公众和新闻舆论监督，大力促进行政权力依法、公正、有效运行。

（三）强化效能意识，增强执行落实的实效性。围绕建设效能型政府，深入推进作风建设年活动，继续开展民主考评市直单位科长活动，着力提升行政效能。大力弘扬雷厉风行、埋头实干的作风，真正把精力凝聚到抓工作、办实事、解难题、求实效上。健全保障落实的工作机制，明确具体目标、时限要求、责任主体，强化执行、反馈、监督、考核，把任务一项一项抓实，把事情一件一件办好。切实改进会风、文风，坚持少开会、开短会，少行文、行短文，少评比、评实效。加强政务督查，强化跟踪问效和行政问责，确保政令畅通。创新行政服务中心管理机制，开辟重大项目并联审批绿色通道，推行企业全程代理服务，切实做到“两集中、两到位”。加大经济环境治理力度，大力整治行政不作为、慢作为、乱作为，严厉打击强装强卸、强买强卖、欺行霸市等扰乱市场秩序的行为。切实加强法制、诚信和人文环境建设，着力营造公平、高效、有序的社会环境，让各类投资者放心创业、放手发展。

（四）强化服务意识，增强亲民为民的主动性。围绕建设服务型政府，结合市、县政府机构改革，加快转变政府职能，深入推进政企分开、政资分开、政事分开、政府与中介组织分开，促使更多的公共资源向农村、基层和困难群众倾斜，为人民群众提供良好的公共服务。充分发挥市长热线电话、电子信箱的作用，支持鼓励网络问政。建立健全领导干部接待群众和工作联系点制度，畅通政府与人民群众的沟通渠道，倾听民声、体察民情，尊重民意、集中民智，充分调动广大人民群众干事创业的积极性、创造性。加强调查研究，坚持工作重心下移，更加关心人民群众尤其是困难群体的生产生活，探索完善各种形式的便民利民措施，着力解决就学、就医、就业和住房等事关人民群众切身利益的问题，满腔热情地为广大人民群众办实事、解难事，让人民群众共享改革发展的成果。

（五）强化廉洁意识，增强拒腐防变的自觉性。围绕建设廉洁型政府，深入推进政治生态建设，大力开展“学习党纪条规、依纪依法办事”主题教育活动，筑牢拒腐防变的思想道德防线。严格落实党风廉政建设责任制，建立健全各项制度，自觉维护制度，认真执行制度，坚决落实制度，真正做到用制度管权、用制度管事、用制度管人，确保权力规范运行、透明运行、阳光运行。加强领导干部经济责任审计，完善政府重大投资项目公示制。加强土地管理、工程建设、环境保护等重点领域的行政监察和审计监督，严肃查处各类违法违纪案件。坚持勤俭办一切事业，积极推进公务消费和公务接待制度改革，大力削减行政成本，把有限财力更多地用在科学发展和民生改善上。全体政府工作人员特别是领导干部要始终牢记“两个务必”，严格遵守廉洁自律各项规定，勤勉尽责、秉公用权，当廉洁奉公的表率，做人民满意的公仆。

各位代表，做好今年的工作，任务艰巨，责任重大。让我们在中共阜阳市委的正确领导下，坚定信心，振奋精神，开拓进取，扎实工作，为科学发展、加速崛起的美好明天而努力奋斗！
